# A p–n Junction by Coupling Amine-Enriched Brookite–TiO_2_ Nanorods with Cu_x_S Nanoparticles for Improved Photocatalytic CO_2_ Reduction

**DOI:** 10.3390/ma16030960

**Published:** 2023-01-19

**Authors:** Zhangjing Chen, Xueteng Zhu, Jinyan Xiong, Zhipan Wen, Gang Cheng

**Affiliations:** 1 School of Chemistry and Environmental Engineering, Wuhan Institute of Technology, Donghu New & High Technology Development Zone, Wuhan 430205, China; 2 College of Chemistry and Chemical Engineering, Wuhan Textile University, Wuhan 430200, China; 3 State Key Laboratory of New Textile Materials and Advanced Processing Technologies, Wuhan Textile University, Wuhan 430200, China

**Keywords:** amine modification, charge separation, p–n junction, photocatalytic CO_2_ reduction, TiO_2_-Cu_x_S composites

## Abstract

Photocatalytic CO_2_ reduction is a promising technology for reaching the aim of “carbon peaking and carbon neutrality”, and it is crucial to design efficient photocatalysts with a rational surface and interface tailoring. Considering that amine modification on the surface of the photocatalyst could offer a favorable impact on the adsorption and activation of CO_2_, in this work, amine-modified brookite TiO_2_ nanorods (NH_2_-B-TiO_2_) coupled with Cu_x_S (NH_2_-B-TiO_2_-Cu_x_S) were effectively fabricated via a facile refluxing method. The formation of a p–n junction at the interface between the NH_2_-B-TiO_2_ and the Cu_x_S could facilitate the separation and transfer of photogenerated carriers. Consequently, under light irradiation for 4 h, when the Cu_x_S content is 16%, the maximum performance for conversion of CO_2_ to CH_4_ reaches at a rate of 3.34 μmol g^−1^ h^−1^ in the NH_2_-B-TiO_2_-Cu_x_S composite, which is approximately 4 times greater than that of pure NH_2_-B-TiO_2_. It is hoped that this work could deliver an approach to construct an amine-enriched p–n junction for efficient CO_2_ photoreduction.

## 1. Introduction

The excessive emission of CO_2_ resulting from the acceleration of industrialization and use of fossil fuel has caused global warming and serious environmental problems [1]. Photocatalytic CO_2_ reduction, denoted as “artificial photosynthesis”, is a promising technology for CO_2_ conversion [2,3,4]. It is critical to develop efficient photocatalysts through surface and interface engineering [5,6]. Semiconductor TiO_2_ has attracted extensive attention concerning CO_2_ photoreduction owing to its stability, low cost, and low toxicity. However, TiO_2_ is an n-type semiconductor with a wide band gap, and the disadvantages of limited light harvesting and poor electron–hole pair separation are not conducive to highly efficient photocatalytic CO_2_ reduction [7,8,9].

Construction of a p–n junction is one promising approach to facilitate the separation of photogenerated carriers and improve the utilization of solar energy [10,11,12,13]. Fan et al. reported that 3D CuS@ZnIn_2_S_4_ p–n heterojunctions with 2D/2D nanosheet subunits can promote the separation of photogenerated carriers and accelerate carrier transfer [14]. Yu et al. suggested that a CuO/TiO_2_ p–n heterojunction can improve the separation efficiency of photogenerated electron–hole pairs [15]. It is well known that CuS and Cu_2_S are p-type semiconductors with narrow bandgaps [16,17]. When coupling Cu_x_S with TiO_2_, upon light irradiation, an internal electric field is established with the formation of a p–n heterojunction. Accordingly, the lower flat band potential of Cu_x_S allows for the transfer of photoexcited electrons from Cu_x_S to TiO_2_, while the holes diffuse from TiO_2_ to Cu_x_S [18,19], which could suppress the charge recombination to achieve an efficient separation of electrons and holes during the photocatalytic process. In this regard, the formation of a TiO_2_/Cu_x_S p–n junction has potential for improving photocatalytic CO_2_ reduction. However, most of the studies upon TiO_2_/CuS p–n junctions focus on the applications of photocatalytic pollution degradation and hydrogen (H_2_) energy generation from water [19,20,21,22]. There are few works relevant to photocatalytic CO_2_ reduction by designing TiO_2_/CuS composites. Recently, Lee et al. [23] reported a CuS_x_-TiO_2_ film could effectively promote photogenerated charge separation for CO_2_ photoreduction. It is still a challenge to rationally design CuS_x_-TiO_2_ p–n junctions for efficient CO_2_ photoreduction.

Due to the unique surface state and higher conduction band position of brookite TiO_2_ compared to anatase and rutile [24,25], great potential has emerged in the field of photocatalytic CO_2_ reduction. Liu et al. reported that defective brookite TiO_2_ had the highest yield for CO and CH_4_ production among the three TiO_2_ polymorphs [26]. Subsequently, Peng’s group studied exposed-crystal-face controlling [27], the construction of heterojunctions [28], and supported metal cocatalysts and dual cocatalysts [29,30] to improve the CO_2_ photoreduction activity of brookite TiO_2_. In fact, the activation and adsorption of CO_2_ are significant factors to enhance CO_2_ photoreduction [31,32,33]. Surface amine modification has attracted great attention in this issue, because the amine groups can not only promote the adsorption and activation of CO_2_, but also coordinate with other metal ions to bind closely. Jin et al. [32] reported that surface amine modification enhances the activity of metal@TiO_2_ photocatalysts. On the basis of the above backgrounds, in this work, amine-modified brookite TiO_2_ nanorods coupled with Cu_x_S nanoparticles has been successfully fabricated. A significant p–n junction is formed between the NH_2_-B-TiO_2_-Cu_x_S interface, which effectively improves the transfer and separation of charge carriers. The composition and morphology of NH_2_-B-TiO_2_ are characterized and the improved performance of photocatalytic CO_2_ reduction is also discussed.

## 2. Experimental

### 2.1. Reagents

All the analytical reagents were used without advance refinement. Tetrabutyl titanate (TBOT), thioacetamide (TAA) and copper (II) acetate monohydrate (Cu(CH_3_COO)_2_·H_2_O) were purchased from Aladdin in China. Ethanediamine (EDA), ethylene glycol (EG) and absolute ethyl alcohol were provided by Sinopharm Chemical Reagent Co., Ltd. (Shanghai, China). All the experiments were conducted with deionized water.

### 2.2. Materials Synthesis

Synthesis of amine-modified brookite TiO_2_ (NH_2_-B-TiO_2_): First, titanium glycolate precursor was synthesized based on our previous reports [34,35,36]; 5 mL tetrabutyl titanate (TBT) was placed into a round-bottomed flask containing 180 mL ethylene glycol (EG) and swirled with magnetic force for 1 h at 120 °C. The white powder material was washed five times with deionized water and once with anhydrous ethanol before being dried at 60 °C for 48 h. To make amine-modified brookite TiO_2_, 0.5 g of the as-prepared titanium glycolate precursor was disseminated in 35 mL of deionized water and 35 mL of ethylenediamine by ultrasonic treatment, and this mixture was uniformly transferred to a 100 mL Teflon-lined stainless-steel autoclave and then placed in an oven at 180 °C for 12 h. Following filtration and washing with deionized water 5 times and with anhydrous ethanol once, finally, the product was gathered after drying at 60 °C for 12 h.

Synthesis of brookite TiO_2_ (B-TiO_2_): Thus method is similar to the method used to prepare amine-modified brookite TiO_2_. Firstly, titanium glycolate precursor was synthesized and then 0.5 g of the as-prepared titanium glycolate precursor was dispersed in 64 mL of deionized water and 6 mL NaOH (1 mol/L) using ultrasonic treatment, which was evenly transferred to a 100 mL Teflon-lined stainless-steel autoclave and later heated at 180 °C for 12 h. Following filtration and washing with deionized water and anhydrous ethanol to pH = 7, the product was gathered and dried at 60 °C for 12 h.

Synthesis of Cu_x_S particles: First, 100 mg Cu(CH_3_COO)_2_·H_2_O (0.5 mmol) was added to a round-bottomed flask containing 20 mL of anhydrous ethanol in an oil bath held at 80 °C under magnetic stirring, to which 42 mg of TAA was added, and kept at reflux for 4 h. The obtained product was centrifuged and washed numerous times with distilled water and anhydrous alcohol.

Synthesis of NH_2_-B-TiO_2_-Cu_x_S: As-synthesized NH_2_-B-TiO_2_ (12.5, 25, 50 mg, respectively) and 10 mg Cu(CH_3_COO)_2_·H_2_O were added to a round-bottomed flask containing 20 mL of anhydrous ethanol in an oil bath held at 80 °C under magnetic stirring, then we added 42 mg of TAA and kept at reflux for 4 h. Then, the suspension was washed with distilled water and distilled alcohol several times via centrifugation. Finally, the obtained product was dried at 60 °C for 12 h. Different ratios of NH_2_-B-TiO_2_-Cu_x_S composites were denoted as NH_2_-B-TiO_2_-Cu_x_S-n, where n represented the molar ratio of Cu_x_S, and the values of n were 8, 16, and 32, respectively.

### 2.3. Characterization of Photocatalysts

X-ray powder diffraction (XRD) measurements were carried out on an Ultima IV X-ray diffractometer with Cu Kα radiation in a range of 10–80° and the scan rate was 10°/min at 40 kV and 30 mA. Fourier-transform infrared (FT-IR) spectra were collected on a Nicolet iS10 IR spectrometer to analyze the chemical bonds and functional groups of the material. Scanning electron microscopy (SEM) and energy-dispersive X-ray spectroscopy (EDX) techniques on a JSM 2100 electron microscope operating at a 200 kV accelerating voltage were used to display the morphologies and elemental dispersion of the samples. X-ray photoelectron spectroscopy (XPS) spectra were measured using Thermo Scientific K-Alpha with an Al Kα X-ray sources (hν = 1486.6 eV, 12 kV 6 mA). All binding energies were calibrated through the C 1s peak at 284.8 eV. UV–vis diffuse reflectance spectrum (UV–vis DRS) was investigated on a Shimadzu UV-2550 spectrometer in a range of 200–800 nm. The photocurrent and electrochemical impedance measurements were performed on a CHI 760E electrochemical workstation, including a standard three-electrode system in 0.2 M Na_2_SO_4_ solution, where Pt wire, Hg/HgCl_2_, and the as-prepared product were used as counter electrode, reference electrode, and working electrode, respectively. Mott–Schottky (M-S) and photocurrent decay plots were also carried out on a CHI 760E electrochemical workstation in 0.2 M Na_2_SO_4_ solution. Among them, the preparation process of the working electrode involved weighing 10 mg of the sample in the sample tube and then adding 1 mL N, N dimethylformamide (DMF) and 20 μL Dupont Nafion membrane solution, which was then stirred for 30 min; after it was completely uniform, we used a pipette gun to transfer 30 μL of the mixed solution coated on the FTO conductive glass. Finally, it was dried in a vacuum oven.

### 2.4. Evaluation of Photocatalytic CO_2_ Reduction

The photocatalytic reduction of CO_2_ was evaluated under irradiation of 300 W xenon lamp and its wavelength was used to simulate sunlight in a gas-closed quartz reactor with a volume of 200 mL. Typically, 50 mg of the as-prepared catalyst was dispersed completely in 1 mL of deionized water in a glass Petri dish, which was then transferred to a quartz reactor with a bottom of 10 mL of deionized water. The reactor was bubbled with high-purity CO_2_ gas for half an hour and the air inside was exhausted prior to illumination. Then, the reaction system was sealed and we turned on the xenon lamp. Subsequently, 1 mL of gaseous product was extracted from the glass reactor by a sampling needle every 1 h, and an irradiation duration of 4 h was applied and analyzed using a gas chromatograph (GC-7900, CEAULIGHT Beijing, China) equipped with an FID detector while N_2_ gas served as the carrier gas. The reactor temperature was maintained at 25 °C and atmospheric pressure after starting the photocatalytic reaction.

## 3. Results and Characterization

**Figure 1:**
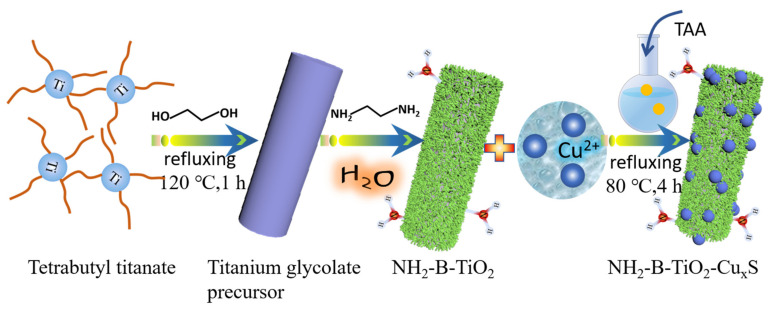
Illustration of the NH_2_-B-TiO_2_-Cu_x_S composites process.

The formation process of Cu_x_S particles supported on amine-modified brookite nanorods is shown in Figure 1. Firstly, the titanium glycolate precursor is synthesized by a simple reflux method. Subsequently, ethylenediamine is introduced to prepare amine- modified brookite TiO_2_ nanorods via a hydrothermal method. Finally, Cu_x_S nanoparticles are deposited on the as-synthesized brookite TiO_2_ nanorods by a refluxing method using copper acetate monohydrate and thioacetamide (TAA) as the precursors. 

**Figure 2:**
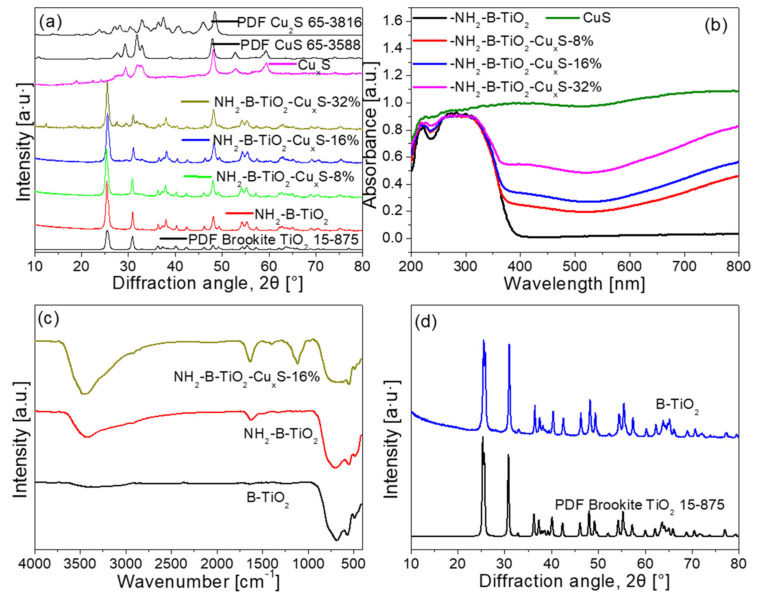
(**a**) XRD patterns of the NH_2_-B-TiO_2_-Cu_x_S composites; (**b**) UV-DRS spectra of the NH_2_-B-TiO_2_, Cu_x_S, and NH_2_-B-TiO_2_-Cu_x_S composites; (**c**) FTIR spectra of B-TiO_2_, NH_2_-B-TiO_2_, and NH_2_-B-TiO_2_-Cu_x_S-16%; (**d**) XRD pattern of brookite TiO_2_ (B-TiO_2_).

Figure 2a shows the X-ray diffraction (XRD) pattern of the as-prepared samples to analyze the composition of the sample. It can be seen that the prepared pure brookite TiO_2_ matches well with the standard one (JCPDF No. 15-875), while the obtained Cu_x_S corresponds to a mixture of the CuS phase (JCPDF No.65-3588) and low-chalcocite Cu_2_S phase (JCPDF No. 65-3816). It can be found that the peaks of brookite TiO_2_ appear in all the as-prepared composites. However, the peaks of Cu_x_S can only be found in the NH_2_-B-TiO_2_-Cu_x_S 32% sample, which may be due to the strong diffraction peaks of brookite TiO_2_ and low contents of Cu_x_S particles in this composite. The UV-Vis absorption (UV-DRS) spectra of the as-synthesized materials are displayed in Figure 2b. It became apparent that the pristine NH_2_-B-TiO_2_ has an absorption edge at around 390 nm. However, the NH_2_-B-TiO_2_-Cu_x_S composites show red shift to the visible light region (400–800 nm), and the light absorption intensity gradually increases with the increase in Cu_x_S loading. Further, the Fourier-transform infrared (FT-IR) is used to analyze the chemical bonds and functional groups of the sample. Figure 2c shows FT-IR spectra of the NH_2_-B-TiO_2_ and NH_2_-B-TiO_2_-Cu_x_S-16% samples, in which the brookite TiO_2_ (B-TiO_2_) (XRD in Figure 2d) is also prepared in the presence of NaOH for comparison. Indeed, the B-TiO_2_ has no amine modification, while the NH_2_-B-TiO_2_ and NH_2_-B-TiO_2_-Cu_x_S samples have two new peaks located at ~1620 and ~3400 cm^−1^, respectively, which may be attributed to the N–H bending vibration and N–H stretching vibration, respectively [37,38].

**Figure 3:**
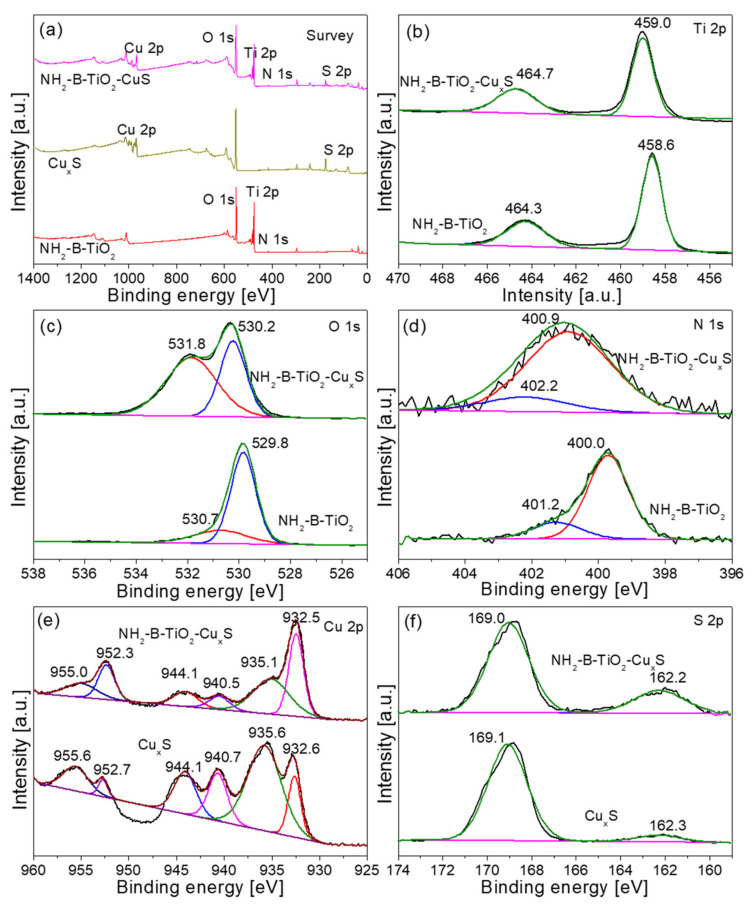
(**a**) XPS survey spectra of NH_2_-B-TiO_2_, Cu_x_S, and NH_2_-B-TiO_2_-Cu_x_S-16% samples; XPS spectra of (**b**) Ti 2p, (**c**) O 1s, (**d**) N 1s, (**e**) Cu 2p and (**f**) S 2p for different materials.

To further confirm the composition of the sample, X-ray photoelectron spectroscopy (XPS) analysis was also performed to study the composition and surface chemical status of samples. Figure 3a shows the full XPS survey spectra of the NH_2_-B-TiO_2_, Cu_x_S, and NH_2_-B-TiO_2_-Cu_x_S samples. Among them, the full XPS survey spectra of the NH_2_-B-TiO_2_-Cu_x_S composite confirms the existence of N, Ti, O, Cu, and S elements. In Figure 3d, for N 1s in NH_2_-B-TiO_2_, the binding energy peak at 400 and 401.2 is attributed to the NH_2_ group and N-H bonds [37,39], respectively, which suggests amine modification was achieved. After loading Cu_x_S, the N 1s peak shifted positively and it was proposed that the amines donate their lone pair of electrons on N atoms to Cu. For NH_2_-B-TiO_2_, the high-resolution XPS spectra of Ti 2p are shown in Figure 3b, and two tiny peaks observed at 458.6 and 464.3 eV are related to Ti 2p_3/2_ and Ti 2p_1/2_, respectively, which indicates the presence of Ti^4+^ [40,41]. Furthermore, Figure 3c exhibits two peaks located at 529.8 and 530.7 eV, which correspond to lattice oxygen and chemisorbed and dissociated oxygen, respectively [42,43,44]. For Cu_x_S, as shown in Figure 3e, the peaks at 932.6 and 952.7 eV belong to Cu 2p_1/2_ and Cu 2p_3/2_ of Cu^+^ species, respectively [45,46], while the peak positions at 935.6 and 955.6 eV are consistent with Cu 2p_3/2_ and Cu 2p_1/2_ of Cu^2+^ [47]. These characteristic peaks with a spin–orbit separation of 20 eV indicate the presence of Cu^2+^ ions [16]. In addition, the other two satellite peaks of Cu^+^ and Cu^2+^ appear at 940.7 and 944.7 eV, respectively. Figure 3f displays the high-resolution spectrum at the S 2p region. The typical peak at 162.3 eV is from S 2p of S^2−^ [48,49]; moreover, a small peak at 169.1 eV is ascribed to SO_4_^2−^, which is due to S^2−^ being oxidized partially [50,51]. However, in the NH_2_-B-TiO_2_-Cu_x_S composite, all the binding energies of Ti, O, and N shift to higher regions, whereas Cu and S shift to lower binding energies compared with the pristine TiO_2_ and Cu_x_S. It might be attributed to chemical interaction and electron transfer in the p–n junction formed at the interface of NH_2_-B-TiO_2_ and Cu_x_S [52]. 

**Figure 4:**
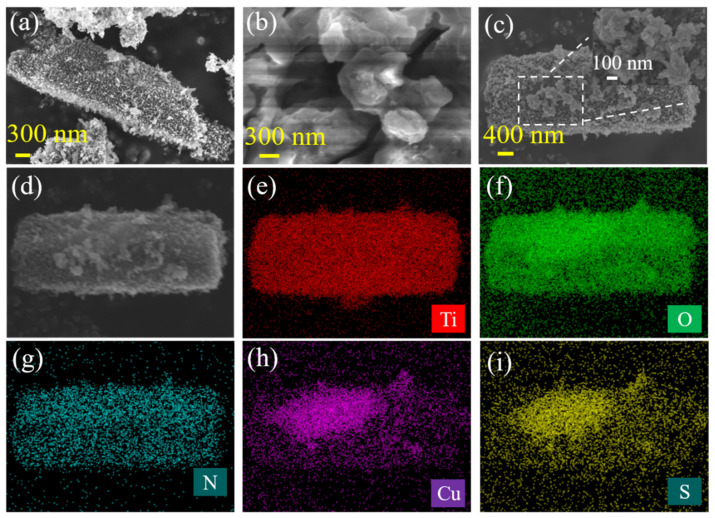
SEM images of (**a**) NH_2_-B-TiO_2_, (**b**) Cu_x_S, and (**c**) NH_2_-B-TiO_2_-Cu_x_S-32% samples; (**d**–**i**) EDX mapping images of the NH_2_-B-TiO_2_-Cu_x_S-32%.

The morphology structures of the as-prepared materials are further determined by SEM. Figure 4a shows that the as-prepared NH_2_-B-TiO_2_ is a rod-like structure with a diameter of 600–800 nm and a length of 2.5–3.5 μm. It can be observed that the microrods assemble with many tiny nanoparticles distributed on the surface. Figure 4b is an SEM image of Cu_x_S, in which the size and morphology are difficult to determine due to the phenomenon of particle aggregation. As shown in Figure 4c, Cu_x_S particles are dispersed on the surface of NH_2_-B-TiO_2_ nanorods. In addition, energy-dispersive X-ray spectroscopy (EDX) mapping images of the NH_2_-B-TiO_2_-Cu_x_S composites show the presence and even distribution of Ti, O, N, Cu, and S elements on the surface of the nanorod in Figure 4d–i, which further confirms that NH_2_-B-TiO_2_ and Cu_x_S are hybridized uniformly.

**Figure 5:**
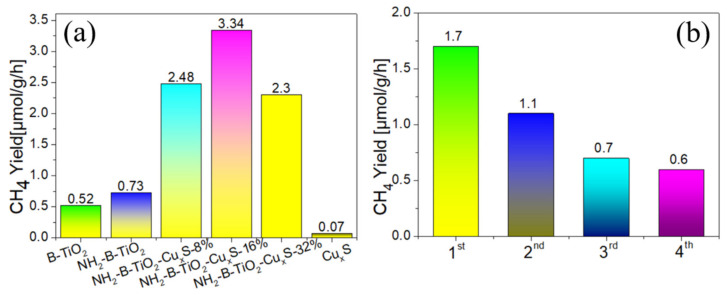
(**a**) Photocatalytic CO_2_ reduction activity of NH_2_-B-TiO_2_, Cu_x_S and NH_2_-B-TiO_2_-Cu_x_S composites. (**b**) Stability of CO_2_ photoconversion in NH_2_-B-TiO_2_-Cu_x_S samples.

In order to explore further CO_2_ photoconversion efficiency, the photocatalytic CO_2_ reduction performances of the as-obtained materials are evaluated in the presence of H_2_O using a gas chromatograph. As shown in Figure 5a, under the irradiation of a 300 W xenon lamp, only CH_4_ was detected in the photocatalytic process of all samples, which may be because the prepared materials only meet the reduction potential of CO_2_ reduction to CH_4_ (E^0^ = −0.24 eV). All the samples have the activity of photocatalytic reduction of CO_2_ into CH_4_, except for the bare Cu_x_S. Among those samples, the brookite TiO_2_ modified with amines (NH_2_-B-TiO_2_) has superior capability to that of B-TiO_2_, indicating that amine modification has a positive effect on the reduction of CO_2_ to CH_4_. With loading of Cu_x_S nanoparticles, the NH_2_-B-TiO_2_-Cu_x_S composites have enhanced performances for photocatalytic CO_2_ reduction into CH_4_. In particular, the NH_2_-B-TiO_2_ shows a low CH_4_ production rate of about 0.73 μmol g^−1^ h^−1^, while the optimized NH_2_-B-TiO_2_-Cu_x_S-16% sample has a yield rate of 3.34 μmol g^−1^ h^−1^, which is 4-fold more than that of the pure NH_2_-B-TiO_2_. Those results suggest that the Cu_x_S could act as a cocatalyst in the amine-enriched B-TiO_2_-Cu_x_S composite for improvements in photocatalytic CO_2_ reduction compared to pristine ones. Typically, to evaluate the stability of the as-synthesized photocatalyst, the photocatalytic CO_2_ reduction activity with five-run cycling of the NH_2_-B-TiO_2_-Cu_x_S 16% sample is tested (Figure 5b). It can be found that the activity of CO_2_ photoconversion to CH_4_ is unstable, and the yield of CH_4_ production gradually decreases, which could be attributed to the deactivation of the composite resulting from the oxidation of Cu_x_S.

**Figure 6:**
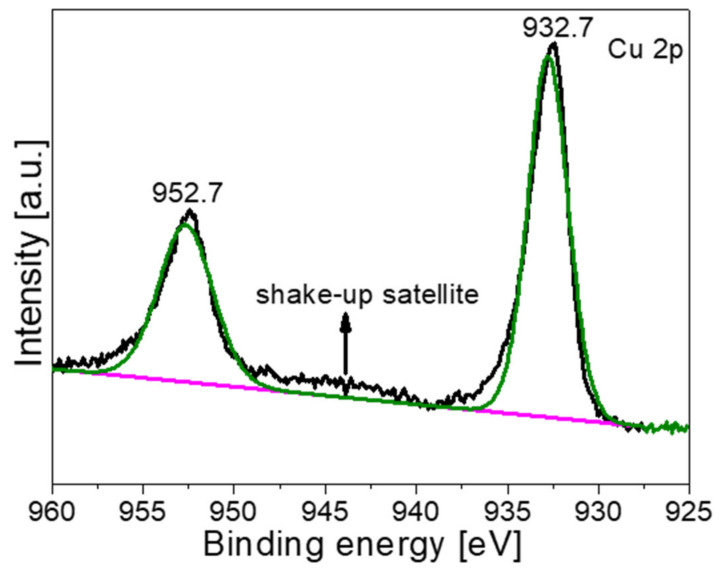
XPS spectra of Cu 2p after the photocatalytic reaction.

Figure 6 shows the XPS spectra of Cu 2p of the NH_2_-B-TiO_2_-Cu_x_S 16% sample after the photocatalytic reaction. It can be observed that the valence state of copper displays obvious changes. The binding energies of 932.7 and 952.7 eV are ascribed to Cu 2p_3/2_ and Cu 2p_1/2_, respectively, a typical peak location of Cu^2+^ in CuS [14,20]. A weak satellite peak around 944 eV further indicates the presence of Cu^2+^ [53]. These results indicate that Cu^+^ was completely oxidized to Cu^2+^ after the photocatalytic reaction in this photocatalysis system, which might weaken the cycling performance of the NH_2_-B-TiO_2_-Cu_x_S composite.

**Figure 7:**
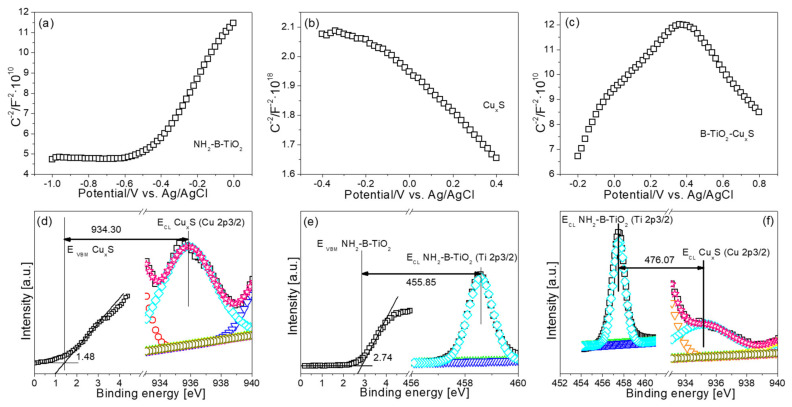
Mott-Schottky plots of (**a**) pure NH_2_-B-TiO_2_, (**b**) Cu_x_S particles, (**c**) NH_2_-B-TiO_2_-16%; VB-XPS and core-level spectrum of (**d**) Cu_x_S and (**e**) NH_2_-B-TiO_2_; (**f**) XPS core-level spectrum of NH_2_-B-TiO_2_-16%.

As a matter of fact, as shown in the Mott–Schottky curve in Figure 7a–c, the slope of NH_2_-B-TiO_2_ is positive, suggesting an n-type semiconductor [54]. At the same time, the slope of Cu_x_S is negative, suggesting that Cu_x_S is an p-type semiconductor. However, the NH_2_-B-TiO_2_-Cu_x_S-16% composite exhibits an inverted “V- shape”, being a symbol of a typical p–n junction [55,56], which indicates that a p–n heterojunction could be constructed between the NH_2_-B-TiO_2_ and Cu_x_S. In addition, the formation of the p–n heterojunction is further confirmed by valence band (VB)-XPS and core-level spectrum analyses. As shown in Figure 7d–f, the band alignment of NH_2_-B-TiO_2_ and Cu_x_S between the NH_2_-B-TiO_2_-Cu_x_S heterojunction interface can be calculated according to the following: Equations (1)–(3) [57].

(1) $$\Delta E_{\mathrm{VBO}}=(E_{\mathrm{CL}}^{{\mathrm{Cu}}_{x}S}-E_{\mathrm{VBM}}^{{\mathrm{Cu}}_{x}S})-(E_{\mathrm{CL}}^{{\mathrm{TiO}}_{2}}-E_{\mathrm{VBM}}^{{\mathrm{TiO}}_{2}})+\Delta E_{\mathrm{CL}}^{\mathrm{Int}}$$

(2) $$∆E_{\mathrm{CL}}^{\mathrm{Int}}=(E_{\mathrm{CL}}^{{\mathrm{TiO}}_{2}}-E_{\mathrm{CL}}^{{\mathrm{Cu}}_{x}S}){\;}^{{\mathrm{NH}}_{2}-B-{\mathrm{TiO}}_{2}-\mathrm{CuxS}-16\%}$$

(3) $$∆E_{\mathrm{CBO}}=E_{g}^{\mathrm{CuxS}}-E_{g}^{{\mathrm{TiO}}_{2}}\;$$

**Figure 8:**
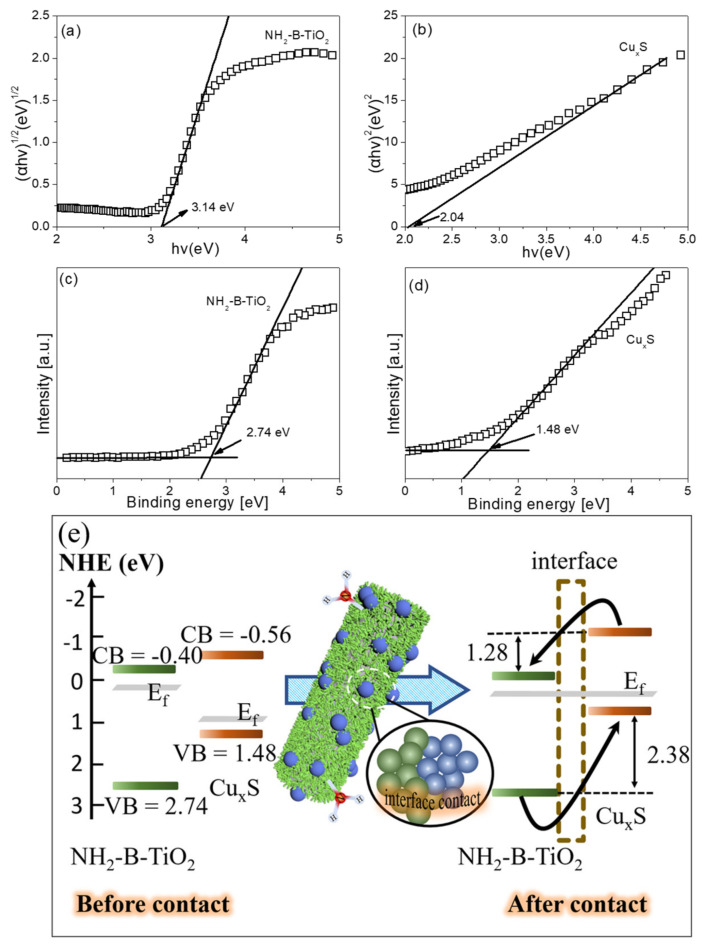
The plot of transformed Kubelka-Munk function of (**a**) NH_2_-B-TiO_2_ and (**b**) Cu_x_S. The valance-band XPS spectrum of (**c**) NH_2_-B-TiO_2_ and (**d**) Cu_x_S; (**e**) schematic illustration for formation of p-n junction on the NH_2_-B-TiO_2_-Cu_x_S composite.

In the above equations, the ΔE_VBO_ represents the valence band offset, which is the energy difference between the core energy level (E_CL_) and the valence band maximum (E_VBM_) in a pure material; meanwhile, $\Delta E_{\mathrm{CL}}^{\mathrm{Int}}$ illuminates the energy difference between the core levels. ∆E_CBO_ represents the conduction band offset. Further, the band gap of the as-synthesized materials is calculated according to the Kubelka–Munk function in Figure 8a,b [58,59], and the band gap energies of pure NH_2_-B-TiO_2_ and Cu_x_S are 3.14 and 2.04 eV, respectively. Based on the information reflected from the XPS and DRS analyses, Figure 8e reveals ΔE_VBO_ = 2.38 eV and ∆E_CBO_ = 1.28 eV for the NH_2_-B-TiO_2_-Cu_x_S nanocomposite. The VB-XPS spectra are verified, as shown in Figure 8c,d, as the valence band position for NH_2_-B-TiO_2_ and Cu_x_S is 2.74 and 1.48 eV, respectively [60]. Therefore, the conduction band position of NH_2_-B-TiO_2_ and Cu_x_S can be calculated as −0.4 and −0.56, respectively. In this regard, the formation of such a p–n junction and the resulting charge transfer are shown in Figure 8e. After contact, the Fermi levels of NH_2_-B-TiO_2_ and Cu_x_S move down and up, respectively, until an equilibrium state is reached. When a built-in electric field between the NH_2_-B-TiO_2_ and Cu_x_S interface is established, this allows for the electrons in Cu_x_S to migrate to NH_2_-B-TiO_2_ while the holes in NH_2_-B-TiO_2_ are transferred to the Cu_x_S. It is proposed that Cu_x_S as a cocatalyst promotes the efficient separation of photogenerated charge carriers.

**Figure 9:**
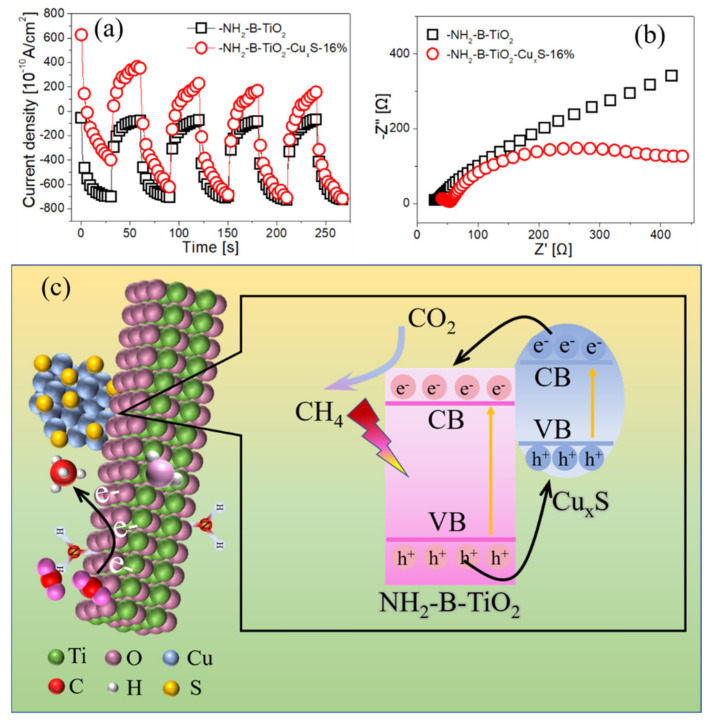
(**a**) Transient photocurrent responses and (**b**) Nyquist plots of NH_2_-B-TiO_2_ and NH_2_-B-TiO_2_-Cu_x_S-16%; (**c**) possible photocatalytic CO_2_ reduction process for the NH_2_-B-TiO_2_-Cu_x_S composite.

Generally, the separation efficiency of photogenerated electrons and holes has a significant impact on the photocatalytic performance [61,62,63]. Herein, photo/electrochemical measurements are carried out to study the charge transfer of the as-synthesized materials. In Figure 9a, the photocurrent density of the NH_2_-B-TiO_2_-Cu_x_S-16% composite is higher than pure NH_2_-B-TiO_2_, which indicates that the loading of Cu_x_S can effectively prevent recombination of the photogenerated electrons and holes. As shown in Figure 9b, the semicircle radius of the NH_2_-B-TiO_2_-Cu_x_S-16% sample is smaller than that of pure NH_2_-B-TiO_2_, implying that the charge carriers have a rapid transfer rate on the composite.

Based on the above results and discussions, a possible CO_2_ photoreduction process is proposed in Figure 9c. Before the photoreduction reaction, the surface amine modification is helpful for the adsorption and activation of CO_2_ [31,37]. Under irradiation of a light source, n-type (NH_2_-B-TiO_2_) and p-type semiconductors (Cu_x_S) generate photogenerated electrons in the conduction band (CB) and holes in the valence band (VB). The CB of NH_2_-B-TiO_2_ is more positive than that of Cu_x_S, and the VB of Cu_x_S is more negative than that of NH_2_-B-TiO_2_. After contact between NH_2_-B-TiO_2_ and the Cu_x_S interface, the built-in electric field is established, which promotes the migration of photoexcited electrons from the CB of Cu_x_S to NH_2_-B-TiO_2_ and the migration of holes from the VB of NH_2_-B-TiO_2_ to Cu_x_S, and these facilitate the separation and transfer of photogenerated electrons and holes. Further, the rate of reduction of CO_2_ to CH_4_ by the photoinduced electrons in CB of NH_2_-B-TiO_2_ is improved.

As shown in Table 1, the CO_2_ photoreduction activity of the NH_2_-B-TiO_2_-Cu_x_S composite is higher than that of other TiO_2_-based binary and ternary composites reported previously.

## 4. Conclusions

In summary, an amine-enriched p–n junction upon the NH_2_-B-TiO_2_-Cu_x_S composite was successfully prepared. The modification of amine on the surface of the photocatalyst has a positive effect on the enhancement of CO_2_ activity. The photocatalytic CO_2_ reduction activity of amine-modified brookite TiO_2_ is higher than that of amine-free modified brookite TiO_2_. Further, coupling different contents of Cu_x_S with NH_2_-B-TiO_2_, the NH_2_-B-TiO_2_-Cu_x_S-16% composite exhibits the greatest CH_4_ yield rate of 3.34 μmol g^−1^ h^−1^ following 4 h of lighting, which is 4 times higher than that of pure NH_2_-B-TiO_2_. Combining the valence band XPS spectra with photo/electrochemical measurements, the formation of a p–n junction between the NH_2_-B-TiO_2_-Cu_x_S interface was confirmed. With the formation of such a heterojunction, the recombination of photogenerated electrons and holes is inhibited, thereby greatly improving the photocatalytic CO_2_ reduction activity. It is hoped that this work could provide an approach to construct amine-enriched p–n junctions for efficient CO_2_ photoreduction. 

## Figures and Tables

**Table 1:** A comparative study on photocatalytic CO_2_ reduction upon different photocatalysts.

Photocatalyst	Light Source	Reaction Condition	CH4 Production Rate [μmol/g/h]	References
NH_2_-B-TiO_2_-Cu_x_S	300 W Xe lamp	H_2_O vapor	3.34	This work
Pt-Cu_2_O/TiO_2_	300 W Xe lamp	H_2_O vapor	1.42	[64]
TiO_2_/g-C_3_N_4_	300 W Xe lamp	H_2_O vapor	2.50	[28]
Au@TiO_2_	300 W Xe lamp	H_2_O vapor	2.52	[65]
CdS/rGO/TiO_2_	300 W Xe lamp	H_2_O vapor	0.063	[66]
Mg-TiO_2_	300 W Xe lamp	H_2_O vapor	1.0	[67]

## Data Availability

All relevant data are contained in the present manuscript.
